# Southern African HIV Clinicians Society clinical guideline for the management of older people with HIV

**DOI:** 10.4102/sajhivmed.v27i1.1809

**Published:** 2026-06-11

**Authors:** Evan Shoul, Nomathemba Chandiwana, Sathya Jogulu, Rene Krause, Coceka Mnyani, Zainab Mohamed, Jeremy S. Nel, Sam Nightingale, Catherine Orrell, W.D. Francois Venter, Camilla Wattrus, Linda-Gail Bekker

**Affiliations:** 1 Netcare Milpark Hospital, Johannesburg, South Africa; 2 Division of Infectious Diseases, Department of Medicine, Faculty of Health Sciences, University of the Witwatersrand, Johannesburg, South Africa; 3 Desmond Tutu HIV Centre, University of Cape Town, Cape Town, South Africa; 4 Kuala Lumpur Primary Care Clinic, Ministry of Health of Malaysia, Kuala Lumpur, Malaysia; 5 Division of Interdisciplinary Palliative Care and Medicine, Department of Family, Community and Emergency Care, University of Cape Town, Cape Town, South Africa; 6 Department of Obstetrics and Gynaecology, School of Clinical Medicine, Faculty of Health Sciences, University of the Witwatersrand, Johannesburg, South Africa; 7 Department of Radiation Oncology, University of Cape Town, Cape Town, South Africa; 8 Groote Schuur Hospital, Cape Town, South Africa; 9 Infectious Diseases and Oncology Research Institute, University of the Witwatersrand, Johannesburg, South Africa; 10 Neuroscience Institute, University of Cape Town, Cape Town, South Africa; 11 HIV and other Infectious Diseases Unit, South African Medical Research Council, Cape Town, South Africa; 12 Ezintsha, Wits Health Consortium, University of the Witwatersrand, Johannesburg, South Africa; 13 Southern African HIV Clinicians Society, Johannesburg, South Africa

## Introduction

The successful global scale-up of antiretroviral therapy (ART) has transformed HIV into a chronic, easily manageable condition. Consequently, the population of people with HIV (PWH) are now also ‘ageing’ with HIV.^1^ Among adults living with HIV in the African region, 15% are aged at least 50 years, and modelling predicts that by 2040 this proportion will increase to 27% (9.1 million).^2^ While ART has expanded the lifespan of PWH,^3^ healthcare workers (HCWs) must now consider how to preserve the ‘health span’ in this group.^4^ This epidemiological shift presents a new set of clinical challenges to clinicians, the health system and societal support structures.

Older people with HIV (OPWH) may experience ‘accelerated’ or ‘premature’ ageing. They face a higher risk of increased burden of non-communicable diseases (NCDs), geriatric syndromes (e.g. frailty, cognitive decline, polypharmacy), and social isolation at an earlier age compared to their HIV-negative peers.^5^ This is because of a combination of chronic inflammation from the ongoing effects of HIV, long-term ART (side) effects, and conventional age-related risks.^6^

This guideline provides a comprehensive, evidence-based and pragmatic framework for the management of OPWH within the South African (SA) healthcare context. It moves beyond viral suppression to advocate for a holistic, person-centred, geriatric-informed approach that prioritises quality of life, functional independence, and the management of multimorbidity. Recommendations are intended for use by HCWs across the spectrum of care, from primary health clinics to tertiary institutions, and in both the public and private sectors. Some of the suggested screening modalities and interventions may be aspirational for the SA public health sector but are included for settings with more resources.

This guideline incorporates principles from the WHO Integrated Care for Older People (ICOPE) framework,^7^ which emphasises prevention prior to frailty, person-centred assessment, and the involvement of HCWs other than doctors. Integrated Care for Older People provides a structured, task-shiftable five-step pathway (Box 1) for detecting and managing declines in intrinsic capacity (IC), defined as ‘the composite of an individual’s physical and mental capacities’. The six IC domains (locomotion, vitality, vision, hearing, cognition, and psychological wellbeing) have been incorporated into the key geriatric syndromes described in this guideline.

BOX 1 Integrated care for older people (ICOPE) five-step pathway for managing intrinsic capacity.

The management of OPWH requires a fundamental shift from a singular focus on HIV virology and the management of a viral infection to a person-centred, multidisciplinary approach that integrates HIV care with geriatric medicine principles.

To enable effective task-shifting and cadre-appropriate care, ICOPE’s five-step pathway can be applied as a structured implementation framework:

1. **Screen:** Briefly (10–15 min) screen for declines in IC domains. *Done by any trained HCW (e.g. nurse, community healthcare worker)*.
2. **Assess:** Conduct an in-depth person-centred assessment to identify impairments and underlying causes. *Done by a nurse, clinical nurse practitioner or doctor, within their scope of practice*.
3. **Develop a care plan**: Create an individualised care plan that addresses identified needs and goals. *Involve the patient and their carers*.
4. **Implement interventions**: Provide appropriate interventions (e.g. rehabilitation, nutrition, social and medical care). *Engage allied HCWs and refer as needed to a specialist for more complex cases*.
5. **Monitor & review**: Regularly follow up, tracking progress and adjusting care. *Engage community resources and include support for carers*.

*Source*: World Health Organization. Integrated care for older people (ICOPE): Guidance for person-centred assessment and pathways in primary care [homepage on the Internet]. 2nd ed. Geneva: World Health Organization; c2024 [cited 2026 May 08]. Available from: https://www.who.int/publications/i/item/9789240103726^7^

ICOPE, integrated care for older people; IC, intrinsic capacity; HCW, healthcare worker.

### The ageing HIV epidemic in South Africa

HIV and the associated chronic immune activation may accelerate the ageing process, leading to an earlier onset of age-associated comorbidities.^6^ There is an increased risk of geriatric syndromes (Box 2) in PWH, including frailty, polypharmacy, cognitive decline, mood disorders, and sensory dysfunction.^6^ This necessitates awareness, proactive screening and intervention at a younger chronological age than would be typical in HIV-negative populations.

BOX 2 Definition of geriatric syndromes.

**Geriatric syndromes** are common conditions in older adults that are multifactorial and don’t fit into distinct organ-based disease categories. They often involve dysfunction across multiple body systems and can lead to functional decline and impact quality of life. These include frailty, polypharmacy, cognitive decline, mood disorders, and sensory dysfunction.

*Source*: Falutz J. Frailty in people living with HIV. Curr HIV/AIDS Rep. 2020;17(3):226–236. https://doi.org/10.1007/s11904-020-00494-2^5^, Akusjärvi SS, Neogi U. Biological aging in people living with HIV on successful antiretroviral therapy: Do they age faster? Curr HIV/AIDS Rep. 2023;20(2):42–50. https://doi.org/10.1007/s11904-023-00646-0^6^

A geriatric assessment provides a complete view of a patient’s function, cognition and health, and improves prognostication and treatment decisions.^8^ As the population with HIV grows older, the application of the geriatric principles can enhance the quality of care.^8,9^

This guidance is designed to:

- Raise HCWs’ awareness of the needs and concerns of PWH who are ≥ 50 years old.
- Inform HCWs about an ageing-related approach to OPWH.
- Highlight good practices to help HCWs provide optimal care for this population.
- Provide resources about ageing with HIV for HCWs, their patients and their patients’ carers.
- Guide clinical settings in implementing geriatric care into HIV clinical practice.

Within the HIV prevention, care and treatment continuum in low- and middle-income countries (LMICs), older people face specific challenges. These are summarised in Table 1.

**TABLE 1:** Specific challenges faced by older people within the HIV continuum of care.

Physiological	Social and behavioural	Structural/health system
Suboptimal immunological response to ART. Some ARVs increase risk of certain NCDs. Increased risk of comorbid conditions (e.g. hypertension, cancer). Increased risk of polypharmacy and its complications, including increased risk of poor adherence, adverse events and DDIs	Perceived low risk of HIV acquisition. Low rates of HIV testing. Low rates of condom use. Low rates of HIV PrEP uptake. Adherence challenges. Continued engagement in risky behaviours. Limited social support.	HCWs unaware of HIV risk among older individuals. Limited availability of differentiated HIV prevention and treatment services. Lack of access to services for non-HIV conditions. Limited mobility restricting access to services. Increased risk of poverty resulting from lack of income.

*Source:* Adapted from The Lancet Healthy Longevity. Ageing with HIV. Lancet Healthy Longev. 2022;3(3):E119. https://doi.org/10.1016/S2666-7568(22)00041-1^10^

ART, antiretroviral therapy; ARVs, antiretrovirals; NCDs, non-communicable diseases; DDIs, drug–drug interactions; PrEP, pre-exposure prophylaxis; HCWs, healthcare workers.

## Principles of care for older people with HIV

An approach to ageing in HIV care should include^11^:

- Discussing the effects of ageing with patients who are ≥ 50 years old and living with HIV, to help identify medical priorities and evaluate physical function. Such conversations may also prompt consideration of advance directives and help patients recognise the effects of age-associated stigma.
- Taking a proactive approach to ageing to help prevent or slow functional and social decline.
- Becoming familiar with the available screening tools, and local and national services for older people.
- Screening for frailty and functional decline to enable early identification of at-risk patients.
- Including nonpharmacologic measures, such as exercise, nutrition and socialisation, which are essential to patients’ physical and emotional health.
- Using a framework such as the ‘geriatric 5Ms’ (mind, mobility, medications, multimorbidity, and matters most) to help inform the choice of screening tests or communicate geriatric concepts. It is also important that screening and assessment be performed with validated tools that assess specific domains.
- Evaluating medications at every clinical visit to eliminate unnecessary or toxic medications, to identify and mitigate potentially harmful drug–drug interactions (DDIs), and reduce the potential for polypharmacy.
- Facilitating and simplifying access to care and services (e.g. aligning appointments and reducing referrals) as patients’ care needs increase to improve overall adherence to and satisfaction with treatment.
- Being familiar with the benefits and local sources of palliative care to help recognise and meet the needs of patients with serious illnesses.
- Enabling task-shifting and cadre-appropriate care in resource constrained settings by applying the ICOPE principles.

The following principles should underpin all clinical interactions with OPWH^11^:

- **Holistic assessment:** Ensuring every consultation extends beyond HIV-specific care and markers (CD4, viral load) to include a review of functional status, mood, cognition, social support, and overall quality of life.
- **Multimorbidity management:** Managing HIV as one of several chronic conditions. Control of hypertension, type 2 diabetes, and other NCDs is equally critical for long-term outcomes.
- **Polypharmacy review:** To minimise adverse effects and interactions, critically reviewing all medications at every visit, including prescribed, over-the-counter, and traditional medicines.
- **Prevention and health promotion:** Prioritising prevention interventions including vaccinations, lifestyle modifications, and screening for geriatric syndromes.
- **Shared decision-making:** Engaging patients and their families, carers and other support structures in all care decisions.
- **Multidisciplinary team approach:** Ensuring the input of a pharmacist, dietitian, physiotherapist, occupational therapist, social worker and mental health professional as required.
- **Intrinsic capacity (IC) monitoring:** Tracking trajectory across the six IC domains (locomotion, vitality, vision, hearing, cognition, and psychological wellbeing) at each visit. A decline in a domain should trigger an in-depth assessment (ICOPE Step 2, Box 1) and individualised care planning (ICOPE Step 3, Box 1).

## Comprehensive health assessment

The Comprehensive Geriatric Assessment (CGA) is valuable, but long and complex. An adapted semi-structured, regular geriatric assessment can be incorporated into primary HIV care for patients ≥ 50 years. Table 2 outlines the minimum recommended screening schedule for all OPWH and integrates ICOPE screening recommendations. The frequency of assessment serves as a guide and should be adapted depending on need and resources. Screening can be conducted by any trained HCW, with more complex assessment or examination, and management undertaken by nurses or doctors within their scope of practice, and with referral to a specialist when needed. Routine HIV care should be provided alongside this.

**TABLE 2:** Screening, prevention, and counselling recommendations for older people with HIV.

Domain	Frequency	Note	Recommended cadre
**History** Corroborate with close family member or carers, when appropriate.			
**Medication** (see sections on polypharmacy, optimising ART)	Every visit	Review of all prescribed, OTC and traditional medications. Ask specifically about calcium, magnesium, iron and St John’s wort, as these can reduce dolutegravir levels. Deprescribe unnecessary medications. Ensure ART is optimised. Check for DDIs using a drug interaction checker (see Box 4).	CNP or doctor (nurse may conduct initial review; deprescribing and DDI management requires CNP or doctor)
**Substance use**	Annually	Screen for harmful alcohol use using AUDIT-C questionnaire (see Box 4). Actively counsel on smoking reduction or cessation and alcohol use. Use a harm-reduction approach.	Any trained HCW
**Mental health** (see section on mental health)	Annually and opportunistically when clinically indicated	Ask about mood, anxiety, functional impairment, and suicide risk. Screen using PHQ-2 and GAD-2 questionnaires for case finding. If positive, proceed to PHQ-9 and GAD-7, and manage actively. See Box 4 for links screening tools.	Any trained HCW (screening); CNP or doctor (management)
**Physical activity** (see section on frailty)	Every visit	Ask about current activity level, physical activity, sedentary time, mobility limitations, pain and history of falls. Ask about difficulty walking or rising from a chair. Encourage reducing sedentary time; any movement is better than sitting, even light activity. If able, aim for at least 150 min or week of moderate activity (e.g. brisk walking), or shorter periods of more vigorous activity. Do strengthening exercises at least twice a week. Include activities that work legs, hips, arms and core. Add balance and functional exercises at least three times a week, especially if risk of falls.	Any trained HCW
**Cognition** (see section on cognitive impairment)	Every visit	Ask about problems with memory and orientation (e.g. recall, date or place). If present, examine.	Any trained HCW
**Sleep** (see section on sleep problems)	Biannually	Ask about sleep quality, insomnia symptoms, snoring or sleep apnoea, daytime sleepiness. Screen for OSA with STOP-BANG; sleepiness with Epworth Sleepiness Scale (see Box 4). Assess and address contributors: mood, pain, substances, ART timing or agents, other medications. Counsel on basic sleep hygiene.	Any trained HCW (screening); CNP or doctor (management)
**Vitality**	Every visit	Ask about unintentional weight loss and reduced appetite. If present, refer for investigation and to dietician for nutritional support, as needed.	Any trained HCW
**Hearing**	Every visit	Ask about difficulty hearing or following conversations, and perform Whisper test.	Any trained HCW (whisper test); refer to audiologist if abnormal
**Vision**	Every visit	Ask about difficulty seeing (even with glasses).	Any trained HCW; refer to optometrist if abnormal
**Sexual health and STIs** (see section on sexual health)	Biannually	Ask about sexual function and libido. Provide contraception as needed. Screen for STIs. If present, examine and manage (see Box 4 for SAHCS STI guideline).	Any trained HCW (history); nurse or CNP or doctor (examination and management)
**Urinary incontinence**	Biannually	Ask about presence and severity. If present, review medication, perform genitourinary examination, and do appropriate blood and urine tests.	Any trained HCW (screening); nurse or doctor (examination and investigations)
**Social support** (see section on psychosocial wellbeing)	Annually	Assess social support.	Any trained HCW; refer to social worker as needed
**Falls history** (see section on falls prevention)	Annually	Ask about falls in the past year.	Any trained HCW
**Driving**	Annually	Discuss safe driving especially if vision, mobility or cognition problems. If problems, recommend stopping driving.	Any trained HCW; CNP or doctor if concerns about cognition, vision or mobility
**Examination**			
**Blood pressure (BP)**	Every visit	If on antihypertensives or reports dizziness, assess for orthostatic hypotension (low BP on standing)	Any trained HCW
**Weight, height and BMI**	Annually	If unintentional weight loss (> 10% per year), assess for sarcopenia, frailty and cancer.	Any trained HCW; dietician
**Frailty** (see section on frailty)	Annually	Assess gait and balance. Perform chair-rise test. Screen for frailty using FRAIL scale (Box 3).	Any trained HCW (FRAIL scale); nurse or CNP (gait, balance, chair-rise test)
**Cognition** (see section on cognitive impairment)	If symptomatic	Screen functionality with IQCODE, or Barthel or Lawton functional assessments. If memory loss or functional decline, assess using MoCA or MMSE. MMSE may be more practical than MoCA (which is not locally validated) but must be interpreted with education level or cultural setting. Perform additional executive function testing if required. See Box 4 for links to assessments.	Nurse or CNP or doctor (choice of test requires expertise; see text); specialist referral if needed
**Renal function** (see section on renal function)	6-monthly	Monitor eGFR. Monitor more frequently if on TDF or with known CKD.	Nurse or CNP or doctor (ordering and interpretation)
**Bone health** (see section on osteoporosis)	Once	Take history of fractures or risk factors. Calculate FRAX score using online calculator (see Box 4) Do DEXA scan in all women > 65 years and men > 70 years, or earlier if high fracture risk based on FRAX score.	Nurse or CNP or doctor (FRAX); DEXA where available (specialist or radiology)
**Lipids**	Annually	Do fasting lipid profile and manage accordingly.	Nurse or CNP or doctor
**Diabetes**	Annually	Do fasting blood glucose or HbA1c, especially if BMI ≥ 25, hypertensive, or hyperlipidaemia.	Nurse or CNP or doctor
**Vaccinations**	*See Table 3 for recommendations*.		Any trained HCW
**Cancer screening**	*See Appendix 1 for recommendations*.		

*Source:* Adapted from Heflin MT. Geriatric health maintenance [homepage on the Internet]. UpToDate. 2025 [updated 2025 Jun 19; cited 2025 Jul 07]. Available from: https://www.uptodate.com/contents/3017^12^ and World Health Organization. Integrated care for older people (ICOPE): Guidance for person-centred assessment and pathways in primary care [homepage on the Internet]. 2nd ed. Geneva: World Health Organization; c2024 [cited 2026 May 08]. Available from: https://www.who.int/publications/i/item/9789240103726^7^

HCW, healthcare worker; CNP, clinical nurse practitioner; OTC, over-the-counter; ART, antiretroviral therapy; DDIs, drug–drug interactions; AUDIT-C, alcohol use disorders identification test-consumption; PHQ-2, patient health questionnaire-2; GAD-2, generalised anxiety disorder 2-item; PHQ-9, patient health questionnaire-9; GAD-7, generalised anxiety disorder 7-item; OSA, obstructive sleep apnoea; STIs, sexually transmitted infections; SAHCS, Southern African HIV Clinicians Society; BP, blood pressure; BMI, body mass index; IQCODE, informant questionnaire on cognitive decline in the elderly; MoCA, Montreal cognitive assessment; MMSE, mini-mental state examination; eGFR, estimated glomerular filtration rate; TDF, tenofovir disoproxil fumarate; CKD, chronic kidney disease; FRAX, fracture risk assessment tool; DEXA, DEXA bone density scan; HbA1c, haemoglobin A1c.

## Immunisations

Age-related decline in immune function is compounded by HIV, leading to suboptimal responses to vaccines and increased susceptibility to vaccine-preventable diseases. Immunisations are a critical part of preventive care among OPWH. Vaccine recommendations are detailed in Table 3.

**TABLE 3:** Immunisation recommendations in older people with HIV.

Vaccine	Who	When
**Influenza** Trivalent or quadrivalent inactivated vaccine	All (especially if chronic lung disease), regardless of CD4 or HIV viral load.	Seasonal: annually between March and May. Pandemic: as required.
**Pneumococcal** PSV23, PCV13	All, if not vaccinated. Can be given if CD4 < 200 cells/µL if on ART with suppressed VL. Poor response if CD4 < 200 cells/µL and VL not suppressed.	Give PCV13 followed 8 weeks later by PPV23. Give booster after 5–10 years. Maximum 2 booster doses, or 1 booster dose if > 65 years.
Zoster*	All ≥ 50 years old, if not vaccinated and resources allow.	Give 2 doses 2–6 months apart.
**Tetanus, diphtheria and pertussis** Tdap	If new grandchildren, pregnant family member/household contact	Give single dose Give booster every 10 years.
**Hepatitis B**	If HBsAg negative	Give 3 doses Give dose 1, then dose 2 at 1 month after dose 1, then dose 3 at 6 months after dose 1

*Source:* Adapted from Dlamini SK, Madhi SA, Muloiwa R, et al. Guidelines for the vaccination of HIV-infected adolescents and adults in South Africa. S Afr J HIV Med. 2018;19(1):1–8. https://doi.org/10.4102/sajhivmed.v19i1.839^13^ and Anderson TC, Masters NB, Guo A, et al. Use of recombinant Zoster vaccine in immunocompromised adults aged ≥19 years: Recommendations of the advisory committee on immunization practices – United States, 2022. MMWR Morb Mortal Wkly Rep. 2022;71:80–84. https://doi.org/10.15585/mmwr.mm7103a2^14^

* Note: At time of publication, the live vaccine (Zostavax^®^) is avaliable in public and private sectors but will soon be discontinued. Recombinant, aduvant vaccine (Shingrix^®^) will soon be registered in South Africa and is currently available through section 21 application.

PSV23, Pneumococcal Polysaccharide Vaccine; PCV13, pneumococcal vaccine 13-valent; ART, antiretroviral therapy; VL, viral load; HBsAg, Hepatitis B surface antigen

## Optimising antiretroviral therapy

If a patient is not yet on ART, start ART at the same visit regardless of their CD4 count. Monitor the viral load response to ART as per the SA national guidelines (Box 4).^15^ Be aware that CD4 recovery may be slower and blunted compared to younger individuals. This highlights the importance of viral suppression as the primary treatment goal.

### Choosing a regimen

The goal is to select an ART regimen that is potent, well-tolerated, has a high barrier to resistance; and has minimal potential for DDIs, and bone and renal toxicities. When choosing a first-line regimen, consider the following:

- Avoid tenofovir disoproxil fumarate (TDF) in patients with, or at high risk for, osteoporosis, bone fractures, or renal impairment (estimated glomerular filtration rate, eGFR < 60 mL/min). Alternative regimens in these patients include:
  ■ **Tenofovir-alafenamide (TAF)-based combination**, for example TAF-emtricitabine-dolutegravir. TAF has a more favourable bone and renal profile than TDF.
  ■ **Abacavir-based regimen**, for example abacavir-lamivudine-dolutegravir. Abacavir is contraindicated in patients positive for HLA-B*5701 and should be used with caution in patients with a high cardiovascular risk.
  ■ **Dual therapy in selected patients:** Lamivudine-dolutegravir is available as a single tablet regimen in the SA private sector. However, it should not be initiated in individuals with a HIV viral load > 500 000 copies/mL, a CD4 count < 200 cells/uL, or those with chronic hepatitis B or any prior history of virological failure. Similarly, rilpivirine-dolutegravir (also available as a single tablet regimen) can be used in patients who are viralogically suppressed without a history of prior virological failure.

For more details on selecting a first-line regimen, opportunistic infection prophylaxis, and renal disease, see https://www.sahivsoc.org/Guidelines/Module11, https://www.sahivsoc.org/Guidelines/Module27 and https://www.sahivsoc.org/Guidelines/Module21, respectively.^16^

### Monitoring on antiretroviral therapy

Age-related declines in renal and hepatic function can alter drug metabolism. Dose selection must be cautious and guided by organ function. It is important to monitor the following:

- **Renal function:** For patients on TDF, monitor creatinine and eGFR at baseline, at 3 months, and then 6-monthly. See https://www.sahivsoc.org/Guidelines/Module15 for more detail.^16^ For details on the recommended baseline evaluation, see https://www.sahivsoc.org/Guidelines/Module7.^16^
- **Weight:** Monitor the patient’s weight trajectory. Significant weight gain on an integrase-strand inhibitor (e.g. dolutegravir) or a TAF-based regimen may require nutritional and lifestyle counselling. Women are at higher risk of weight gain.
- **Adherence:** OPWH may have better adherence than younger cohorts, but screen for barriers related to polypharmacy, cognitive decline, and mental illness.

### Managing adverse effects on antiretroviral therapy

Neuropsychiatric and metabolic side effects should be monitored:

- If a patient on efavirenz or dolutegravir reports significant insomnia, mood changes, or depression, switch to an alternative agent (e.g. rilpivirine in virologically suppressed patients, or a protease inhibitor [PI]-based regimen).
- Manage weight gain, dyslipidaemia, and glucose intolerance proactively with lifestyle and pharmacological interventions. Avoid unnecessary ART switches.

## Geriatric syndromes

### Frailty

Frailty increases vulnerability to stressors and is a powerful predictor of negative health outcomes in older people. To screen for frailty, use the FRAIL questionnaire (Box 3). A score ≥ 3 indicates frailty.

BOX 3 FRAIL questionnaire.

**The five FRAIL questions** (≥ 3 points indicates frailty):

**Fatigue:** ‘How much of the time during the past 4 weeks did you feel tired?’ (Yes to ‘all’ or ‘most of the time’ = 1 point).

**Resistance:** ‘By yourself and not using aids, do you have any difficulty walking up 10 steps without resting?’ (Yes = 1 point).

**Ambulation:** ‘By yourself and not using aids, do you have any difficulty walking several hundred yards?’ (Yes = 1 point).

**Illnesses:** ‘Do you have ≥ 5 of these 11 illnesses?’ (hypertension, diabetes, cancer, chronic lung disease, heart attack, heart failure, asthma, arthritis, stroke, kidney disease) (Yes = 1 point).

**Weight Loss:** ‘Have you lost > 5% of your body weight in the past year?’ (Yes = 1 point).

*Source:* Morley JE, Malmstrom TK, Miller DK. A simple frailty questionnaire (FRAIL) predicts outcomes in middle aged African Americans. J Nutr Health Aging. 2012;16(7):601–608. https://doi.org/10.1007/s12603-012-0084-2^17^

Manage frailty as follows:

- **Help the patient to develop an exercise plan:** Supervised resistance and balance training are most effective. Refer to a physiotherapist or occupational therapist if necessary and if resources allow.
- **Assist with good nutrition:** Ensure adequate protein and calorie intake to prevent sarcopenia. Refer for nutritional support if necessary and if resources allow.
- **Optimise comorbidities:** Ensure good control of NCDs.
- **Review medications:** Deprescribe medications that contribute to fatigue, weakness or dizziness.
- **Assist with social support:** Address isolation and lack of support. Refer to a social worker if necessary and if resources allow. Also see Box 4.

BOX 4 Useful resources for clinicians.

- ICOPE handbook: Integrated care for older people (ICOPE): guidance for person-centred assessment and pathways in primary care, 2nd edn
- DementiaSA: https://dementiasa.org/.
- Home Base Care organisations and Hospice support: https://palprac.org/ and https://apcc.org.za/.
- Liverpool website to check DDIs: https://hiv-druginteractions.org/checker
- PHQ and GAD questionnaires: Generalized Anxiety Disorder 2-item (GAD-2) - Mental Health Screening – National HIV Curriculum
- SPICT-SA tool: Version-2-SPICT-SA.pdf
- STOP-BANG Questionnaire: The Official STOP-Bang Questionnaire Website
- Epworth Sleepiness Scale: Epworth Sleepiness Scale (ESS) Screening Tool | Sleepcountshcp
- AUDIT-C questionnaire: Form: AUDIT-C Questionnaire
- FRAX score online calculator: https://www.fraxplus.org/calculation-tool/
- Framingham score: https://www.mdcalc.com/calc/38/framingham-risk-score-hard-coronary-heart-disease
- FRAIL questionnaire : https://efrailty.hsl.harvard.edu/index.html
- Tools for cognitive testing:
  ■ Montreal Cognitive Assessment (MoCA): | MoCA Test
  ■ Mini Mental State Examination (MMSE): Mini-Mental State Exam (MMSE) Test for Alzheimer’s / Dementia
  ■ Addenbrooke’s Cognitive Examination (ACE-III): https://www.rightdecisions.scot.nhs.uk/media/ztyhfgfj/ace-iii-2017-uk-version-a.pdf
- Southern African HIV Clinicians Society guidelines:
  ■ Sexually transmitted infections: SAHCS 2022 STI guidelines.pdf (SECURED)
  ■ Syphilis: SAHCS syphilis guideline.pdf
  ■ ART: Southern African HIV Clinicians Society
  ■ PrEP: SAHCS PrEP guideline 2025.pdf
- SA National Department of Health guidelines:
  ■ Consolidated ART guidelines: National Consolidated Guidelines 04-03-2026.pdf
  ■ Standard Treatment Guidelines for Primary Health Care (STG/EML): PHC-Standard-Treatment-Guidelines-and-EML-8th-Edition-2024.pdf
  ■ National Guidelines on the Treatment of Tuberculosis Infection: Health_Latent%20TB%20Infection_2023_web.pdf
  ■ Clinical Management of Rifampicin-Resistant Tuberculosis: Updated RR-TB Clinical Guidelines September 2023.pdf

### Polypharmacy

Polypharmacy is the use of ≥ 5 medications. It is common among OPWH and a major driver of adverse drug events, non-adherence, and hospitalisations.^18,19^ Managing polypharmacy is challenging and ideally the primary carer should regularly review all medication.

Where a medication is indicated, a good general rule in OPWH is to start with a low dose and titrate the dose upwards moderated by response and side effects.

Manage polypharmacy as follows:

- Create a complete an accurate list of all medications, including prescription, over-the-counter and traditional medicines.
- Identify potentially inappropriate medications (e.g. sedatives, anticholinergics, duplicate drug classes).
- Check for DDIs for every new drug prescribed using an interaction checker (Box 4).
- Taper and stop any non-essential medications and where risks outweigh benefits.^20^
- Simplify prescribed medication by using combination pills (e.g. single-pill ART, fixed-dose combinations) to reduce pill burden.

### Cognitive impairment

HIV itself may cause cognitive impairment and OPWH are at higher risk for all-cause dementia.^21,22^ Causes of cognitive impairment include those caused directly by HIV (HIV-associated brain injury [HABI]) and those caused by comorbid factors (Table 4).^22^ Progressive cognitive impairment caused directly by HIV (active HABI) tends to be associated with incomplete HIV control in the plasma and/or cerebrospinal fluid (CSF), whereas neurodegenerative diseases (such as Alzheimer’s disease) can occur in OPWH with HIV suppression.

**TABLE 4:** Causes of brain injury in people living with HIV.

HIV-associated brain injury (HABI)	Other causes of brain injury
Legacy HABI: Inactive brain injury from pre-ART damage. Active HABI: Ongoing brain injury leading to clinical or radiological progression	Neurological causes: ■ Neurodegenerative disorders (e.g. Alzheimer’s disease) ■ Cerebrovascular disease ■ Developmental disability ■ Traumatic brain injury ■ Non-HIV related neurological conditions (e.g. multiple sclerosis or uncontrolled epilepsy) ■ Nutritional deficiencies (e.g. vitamin B12) Infection related: ■ CNS infections (e.g. TB, cryptococcosis, neurosyphilis) ■ Coinfections (including syphilis and hepatitis C) Toxin related: ■ Hazardous alcohol use ■ Substance misuse ■ Antiretroviral CNS neurotoxicity (e.g. efavirenz)

*Source:* Adapted from Nightingale S, Ances B, Cinque P, et al. Cognitive impairment in people living with HIV: Consensus recommendations for a new approach. Nat Rev Neurol. 2023;19(7):424–433. https://doi.org/10.1038/s41582-023-00813-2^23^

HIV, human immunodeficiency virus; HABI, HIV-associated brain injury; ART, antiretroviral therapy; CNS, central nervous system; TB, tuberculosis.

Older people with HIV should be encouraged to report issues with memory, concentration and functioning, and any concerns should prompt assessment. This should be corroborated or supplemented by an informant, such as a carer or family member, as appropriate. Routine screening using a cognitive tool in *asymptomatic* people is not recommended.

Choice of cognitive test will depend on time and available expertise. Commonly used tools include the Montreal Cognitive Assessment (MoCA), the Mini Mental State Examination (MMSE), and the Addenbrooke’s Cognitive Examination (ACE-III) (see Box 4). These tools have been developed in the global north, and cut-offs for abnormality do not apply across all populations. Results should therefore be interpreted in the context of the individual’s educational and sociodemographic background. The International HIV-Dementia Scale has low sensitivity and is no longer recommended.

Assess cognitive impairment as follows:

- Ideally include an informant (e.g. a relative or carer) when taking a history, as cognitively impaired people may lack insight into their difficulties. Confidentiality should be preserved and consent obtained.
- Screen with a functional assessment (tools include IQCODE, Barthel, Lawton). See Box 4 for links to tools.
- Functional assessment can also predict dementia stage when cognitive testing is unreliable.
- Assess for depressive symptoms as these can contribute to cognitive symptoms and/or confound cognitive testing.

Perform investigations depending on available resources:

- Exclude reversible causes including hypothyroidism (thyroid stimulating hormone, TSH), vitamin B12 deficiency (serum B12), and syphilis (TPHA).
- Consider neuroimaging where resources permit: Computerised tomography (CT) brain imaging can indicate comorbid pathology including cardiovascular disease (CVD), traumatic brain injury or previous opportunistic infection. Magnetic resonance imaging (MRI) may additionally show diffuse white matter signal changes indicating legacy HABI, or focal cortical atrophy indicating a neurodegenerative process such as Alzheimer’s disease.
- Perform a lumbar puncture only in those with suspected active HABI. This should include measurement of CSF HIV viral load and investigating possible central nervous system infections, such as neurosyphilis.

Management of cognitive impairment:

- Ensure sustained HIV viral suppression.
- Optimise vascular risk factors: control hypertension, diabetes, and hyperlipidaemia.
- Think of and treat comorbidities: depression/psychiatric illness, delirium, alcohol, seizures.
- Avoid ART neurotoxicity: If on efavirenz, switch ART as this is associated with cognitive side effects, which can be severe.^24^
- Avoid sedatives and anticholinergics including tricyclic antidepressants.
- If needed, offer information and practical support.^25^ Refer to social worker if necessary and if resources allow.

### Falls prevention

It is important to screen OPWH for fall risk. Use a simple physical performance test such as:

- Timed-Up-And-Go (TUG)^26^:
  ■ Ask the patient to stand up from chair, walk 3 m, turn, walk back and sit down.
  ■ *Results:* < 10 s normal; >14 s increased fall risk; > 20 s frailty or mobility impairment.
- 5 times sit to stand (5 x STS)^27^:
  ■ Ask the patient to sit on chair, fold their arms, stand up and sit down five times.
  ■ *Results:* > 15 s fall risk; > 20 s or unable is frailty.

Evaluate for causes of fall risk including:

- **Medications:** sedatives, antidepressants, antihypertensives.
- **Environmental:** home hazards, poor lighting, loose rugs.
- **Medical:** orthostatic hypotension, visual impairment, neuropathy, foot problems.
- **Neuromuscular:** weakness (sarcopenia), poor balance.

Managing falls risk includes exercise programmes (balance and strength training), medication review and management of orthostatic hypotension. If resources allow, physiotherapist or biokineticist referral is recommended for those at high risk. Self-exercise is recommended for those at low risk. If resources allow, consider referral to an occupational therapist for home safety modifications and refer to podiatrist as needed.

## Mental health and psychosocial wellbeing

Depression, anxiety, and substance use disorders are common in OPWH, and are associated with reduced quality of life, poor ART adherence, functional decline, and increased morbidity.^28,29,30^ These conditions are worsened by social and structural stressors, stigma, loneliness, poverty, food insecurity, and the demands of managing multiple comorbidities.^31,32^

Certain antiretrovirals have been associated with neuropsychiatric symptoms in susceptible individuals and should be reviewed in the context of new or worsening symptoms. Efavirenz remains a commonly used non-nucleoside reverse transcriptase inhibitor (NNRTI), despite evidence of neuropsychiatric side effects such as sleep disturbances, suicide, depression and neurocognitive effects.^20^ Dolutegravir use in OPWH has also been associated with sleep and mood alterations, anxiety, depression, and psychosis.^20^

Routine mental health screening should be integrated into routine HIV care and done at a primary care level:

- Annual screening is recommended with brief primary healthcare tools such as the Patient Health Questionnaire-2 (PHQ-2) for depression and Generalised Anxiety Disorder-2 (GAD-2) for anxiety. Should these screen positive, more detailed tools such as PHQ-9 and GAD-7 should be used. These are effective across all resource settings.^33,34^ See Box 4 for links to tools.
- Always assess for suicidal ideation in patients with depression. This is a psychiatric emergency that if present requires immediate referral.

Management includes^35^:

- **Exclude underlying medical conditions and optimise treatment of chronic comorbidities**, such as hypothyroidism, anaemia, tuberculosis (TB), diabetes
- **Screen for and manage substance use**, for example alcohol use, over-the-counter analgesics.
- **Optimise first-line pharmacotherapy:** use a selective serotonin reuptake inhibitor (SSRI) such as fluoxetine or citalopram and ‘start low, go slow’ or Serotonin-Noradrenaline Reuptake Inhibitors (SNRIs) such as duloxetine, especially in HIV patients with sensory neuropathies. Titrate to clinical response which can take up to 8 weeks. Be aware of potential DDIs with ART, for example, ritonavir. Use an interaction checker (Box 4).
- **Refer for psychotherapy:** refer for individual therapy and/or family counselling if available. The ‘Friendship Bench’ model is an effective and affordable task-shifted intervention which relies on peer or lay support in a community-based setting.
- **Assist with social support:** facilitate access to support groups, community clubs, and social grants to address isolation and poverty.
- For more information see Chapter 16 of the National Standard Treatment Guidelines for Primary Health Care (Box 4).^35^

## Managing comorbid non-communicable diseases

### Cardiovascular disease

Older people with HIV on ART have double the risk for myocardial infarction and heart failure when compared with the general population. Along with traditional risk factors, heightened inflammation and ART toxicities contribute to these risks.^36^ Certain antiretrovirals (e.g. abacavir, lopinavir-ritonavir), though not others (e.g. atazanavir), are associated with increased risk of myocardial infarction.^37^

Approaches to metabolic and cardiovascular risk are similar to those for the general population, with the following caveats:

- For risk calculation, use a standard CVD risk calculator, for example the Framingham score (see Box 4), acknowledging that it may underestimate risk in PWH.
- Modifiable risk factors warrant close attention, including smoking, diabetes, chronic kidney disease, substance use, depression, and hepatitis C.
- Avoid simvastatin and lovastatin if on PI-based ART because of DDIs. Preferred options are atorvastatin or rosuvastatin, with appropriate dosing.
- Abacavir has been inconsistently linked to elevated cardiovascular risk. Assess individualised risks and benefits. Tenofovir-containing regimens are preferred where possible.
- Low dose aspirin as primary prevention in older adults results in higher risk of major haemorrhage with no lower risk of CVD, and should therefore not be used.^38^

### Obesity

People with HIV are at increased risk of obesity as they age, especially with the use of certain antiretrovirals including the integrase strand transfer inhibitors (INSTIs) and TAF. While all ART and HIV itself cause weight gain, the underlying cause is not well understood. Common risk factors include advanced disease at ART initiation (low CD4/high viral load, low body mass index [BMI]), women, Black ethnicity, TAF versus TDF, and INSTIs versus PI- or efavirenz-based regimens.

Lifestyle interventions have limited long-term benefit, while newer obesity medications show promise. Research on new drugs in PWH is sparse and access is constrained by cost, availability, and provider expertise.

Obesity prevention strategies at ART initiation should focus on culturally and contextually appropriate counselling around diet and activity. Switching ART is generally not advised, except possibly from TAF to TDF, which may modestly reduce weight gain but must be balanced against renal and bone health risks.

### Diabetes

Diabetes screening in OPWH should follow local guidelines and management, recognising that metformin and dolutegravir have a DDI, and a dose of 1g twice daily (total daily dose of 2 g) of metformin should not be exceeded.

### Hypertension

Hypertension screening and management remains conventional for OPWH, with the only consideration being possible DDIs between the PIs and calcium-channel blockers, which may mean extra monitoring of hypotension side effects, and avoidance of nifedipine.

### Osteoporosis

Osteoporosis is common in PWH because of multiple risk factors, including low BMI, hypogonadism, smoking, and the long-term effects of HIV infection and ART. This leads to higher fracture risk, morbidity, and mortality in OPWH.^39^ Tenofovir disoproxil fumarate has been linked to significant bone mineral density (BMD) loss and higher fracture risk, and switching to TAF may reduce this toxicity.^20^ Diagnosis is based on BMD measurement or a history of fragility fracture (vertebral, proximal femur, distal radius).

Screening for osteoporosis can be done as follows:

- A fracture risk assessment tool (FRAX) calculation (can be done without BMD result) and can be used as a simple screen even in low resource settings. Check ‘yes’ for secondary causes in PWH. If fracture risk is high, then refer for BMD screening. See Box 4 for a link to the tool.
- If resources allow, perform bone density screening using a DEXA scan for all women > 65 years and men > 70 years, or earlier in those with risk factors (e.g. steroid use, prior fracture, prolonged TDF exposure).

Management of osteoporosis should include^39,40,41^:

- General measures:
  ■ Basic investigations can be performed to exclude secondary causes, such as calcium, parathyroid hormone (PTH), alkaline phosphatase (ALP), albumin, creatinine, full blood count (FBC) with erythrocyte sedimentation rate (ESR), TSH, serum protein electrophoresis (SPEP).
  ■ Encourage healthy eating, adequate calcium and vitamin D intake, and regular physical activity.
  ■ Advise the patient to limit alcohol and stop smoking.
  ■ Give supplemental calcium (500 mg/day – 600 mg/day) and vitamin D (600 IU/day – 800IU/day). Calcium supplements can reduce dolutegravir absorption. Advise patients to take calcium with meals and at least 2 h before or after dolutegravir.
  ■ Institute fall prevention strategies.
- ART switch:
  ■ Consider switching from TDF to TAF or abacavir in patients with osteoporosis or fragility fractures.
- Pharmacotherapy
  ■ Consider hormone therapy for post-menopausal women: oestrogen plus progestin
  ■ Give a bisphosphonate (first-line for most patients): ibandronate (orally or intravenously), alendronate orally (daily/weekly), zoledronate (annually, intravenously).
    ◦ If medications are not tolerated or there is a poor clinical response, refer for specialised second line treatment.
    ◦ Recommend ‘treatment holidays’ from bisphosphonates: after 5 years of alendronate, and 3–6 years of zoledronate, and consider treatment holiday with ibandronate after 5 years if fracture risk is low.

### Renal function

Renal disease in OPWH can result from HIV infection itself (local infection and immune dysregulation), ART-related renal toxicity, genetic susceptibility and comorbid chronic kidney disease (CKD) risk factors such as diabetes, hypertension, and hepatitis B and C.

Age-related decline in renal function alters the pharmacokinetics of nucleoside reverse transcriptase inhibitors (NRTIs) such as TDF, lamivudine and emtricitabine.^20^ As eGFR declines, NRTI doses should be adjusted as titrated against renal function. NNRTIs, PIs and INSTIs do not require renal adjustment. Risk factors for renal toxicity associated with TDF include poor baseline renal function, concomitant nephrotoxic medications, low BMI, older age and lower CD4 count.^20^

Managing renal function should include:

- Monitor eGFR 6-monthly.
- Avoid nephrotoxic drugs (e.g. nonsteroidal anti-inflammatory drugs [NSAIDs], aminoglycosides) in patients with CKD.
- Dose-adjust lamivudine, emtricitabine, and TDF according to eGFR.
- TAF is preferred over TDF in CKD.
- If the patient is persistently proteinuric or dialysis is required, discuss and co-manage with a nephrologist.

For more detail, see www.sahivsoc.org/guidelines/Module21.^16^

### Hepatic function

Many drugs used in PWH are metabolised by the liver. Age-related decline in liver function results in increased drug exposure with increased risks of adverse effects. Older people with HIV may present with more severe liver disease, particularly co-infection with Hepatitis B or C, metabolic disorders such as metabolic dysfunction-associated steatotic liver disease and hepatocellular carcinoma.^20^

The least hepatotoxic first line regimen is TDF (or TAF) plus lamivudine (or emtricitabine) plus dolutegravir. For individuals with advanced liver disease (Child-Pugh class C), PIs and NNRTIs are best avoided. If their use is essential, closely monitor liver function for the first 3–6 months. Abacavir, which undergoes extensive hepatic metabolism, requires a dose reduction in the setting of advanced liver disease^42^:

- Mild impairment (Child-Pugh Class A): 200 mg 12-hourly
- Moderate and Severe impairment (Child-Pugh Class B and C): contra-indicated.

Other common medications with a risk of hepatoxicity include cotrimoxazole and anti-tuberculous drugs, including tuberculosis preventive therapy (TPT) regimens. These medications should not be unnecessarily withheld in OPWH because of their clinical benefits. However, for individuals with pre-existing severe liver disease, an individualised risk-benefit assessment is essential, as such medications may not be appropriate in this context.

Patients with chronic hepatitis B require a tenofovir-based regimen (TDF or TAF).

For more information see: https://www.sahivsoc.org/Guidelines/Module20^.16^

### Lung health

#### Asthma and chronic obstructive pulmonary disease

Management of asthma and chronic obstructive pulmonary disease (COPD) in OPWH remains similar to the general population except when considering potential DDIs between ART and asthma medications. Use of PIs with inhaled or systemic corticosteroids and long-acting inhaled beta agonists may cause added toxicities in the asthma drugs and should be closely monitored.^43,44^ Assess cumulative systemic corticosteroid exposure, as it may worsen age-related comorbidities, particularly osteoporosis.

#### Tuberculosis

Older people with HIV are at increased risk for both primary TB and reactivation of latent TB infection. Older people are more likely to develop extra-pulmonary and atypical forms of TB that are often harder to diagnose than conventional smear-positive pulmonary TB.^45^ Prevention, screening and treatment of TB should follow National guidelines (Box 4), with the added caution of DDIs between rifampicin and ART (dolutegravir and PIs). Patients on TLD require an additional 50mg dolutegravir twice daily while on rifampicin. OPWH are more susceptible to adverse effects from TB treatment and require closer follow up.^45,46,47^

### Sleep problems

Sleep disturbances are common in OPWH and significantly impact mental health, neurocognitive functioning, cardiometabolic risk and overall functional status.^48,49,50^ Poor sleep quantity and quality worsens anxiety, chronic pain, depression, and cognitive decline, while also increasing the risk of type 2 diabetes and hypertension.^48,51,52,53,54^

Screening for sleep problems includes:

- Routinely enquire about sleep quantity, quality, timing, daytime sleepiness, and functional impact, with collateral history where appropriate.
- Use brief screening tools (Box 4) to identify common sleep problems, including the Epworth Sleepiness Scale (daytime sleepiness) and STOP-BANG (risk of obstructive sleep apnoea).
- Consider contributory factors, including depression, anxiety, chronic pain, alcohol use, ART-related effects, and polypharmacy.

Management should follow a stepwise approach, addressing reversible contributors before specialist referral:

- Identify and treat contributing conditions, including depression, anxiety, chronic pain, alcohol or substance use, cardiometabolic disease, and poorly controlled HIV.
- Review ART and concomitant medications in individuals with persistent or worsening sleep disturbance.
- Prioritise non-pharmacological approaches, including sleep hygiene education, physical activity, and cognitive behavioural therapy for insomnia (CBTi) where available.
- Refer for diagnostic sleep studies and specialist care if resources allow, particularly when obstructive sleep apnoea is suspected.
- Avoid the routine use of benzodiazepines, benzodiazepine receptor agonists (e.g. zolpidem, zopiclone), and tricyclic antidepressants for insomnia in older adults, because of increased risk of falls, cognitive impairment, and other adverse events.^55^
- Where pharmacological treatment is considered, use the lowest effective dose for the shortest duration, with close monitoring for adverse effects and DDIs.
- In selected cases with suspected circadian rhythm disruption, chronotherapy (timed light exposure and/or melatonin) may be considered in specialist or well-supported settings.

## Sexual and reproductive health

Sexual and reproductive health (SRH) needs in older people are often neglected. While condom use declines with age, sexual activity continues, leading to increased sexually transmitted infection (STI) risk. The risk of STIs, including HIV, is often not considered in older people, delaying diagnosis and initiation of treatment.^56,57^

### Women

Sexual health is impacted by menopause and its physiological and psychological symptoms. Menopause changes the vaginal microbiota, increasing the risk of HIV transmission and acquisition.^58^ Hormone therapy can be considered after an individualised risk–benefit assessment. Contraception remains necessary until menopause is confirmed, defined as at least 12 months of amenorrhoea. The choice of contraception should be guided by existing comorbidities.

### Men

Common sexual dysfunctions in older men include erectile dysfunction, ejaculatory disorders, and primary testicular failure. Treatment options for erectile dysfunction range from lifestyle modifications and medical management to oral phosphodiesterase type-5 inhibitors (PDE5i), such as sildenafil. While PDE5 inhibitors are effective, they have potential DDIs with nitrates and some ART. Dose adjustments of PDE5 inhibitors may be required if given with PIs.^59^

## Malignancies

Screening for malignancy is essential in OPWH because of increased cancer risk from ageing, longer survival on ART, and premature ageing in PWH.^60^

People with HIV are at increased risk of AIDS-defining cancers (e.g. Kaposi sarcoma, non-Hodgkin lymphoma, and cervical cancer) and non-AIDS-defining cancers (e.g. Hodgkin lymphoma; anal, lung, liver, and oropharyngeal cancers).^61,62^

Appendix 1 details cancer screening recommendations in OPWH.

Strategies to reduce cancer risk include:

- **Lifestyle modifications:** Smoking cessation, reduced alcohol intake, obesity management, healthy diet and regular physical activity.^61,63,64,65,66^
- **Vaccinations**: Human papillomavirus (HPV) vaccine prevents cervical carcinoma and other HPV-related malignancies. Screening and vaccination for Hepatitis B in OPWH as well as early treatment initiation can prevent hepatocellular carcinoma.^61,67,68,69^

Managing cancer in OPWH includes:

- **Coordinate ART and oncotherapy**: Assess and manage potential drug interactions between ART and cancer treatments (chemotherapy, targeted therapy, etc.).
- **Monitor immunotherapy use:** Evaluate the safety and efficacy of immunotherapy in the context of HIV, considering immune status and response.
- **Address age-related toxicity**: Monitor and manage increased treatment-related toxicity as a result of age, especially in older patients with HIV.
- **Track immune and viral status:** Regularly monitor CD4 cell counts and HIV viral load, as these may influence cancer treatment choices and outcomes.
- **Manage comorbidities:** Assess and manage cardiovascular and kidney disease, which can affect cancer treatment decisions and patient tolerance.^70,71^

## Palliative and end-of-life care

### Palliative care

Palliative care aims to enhance the quality of life for patients and their families who have been diagnosed with life-threatening illnesses. Ageing accompanied by certain incurable diseases should necessitate the transition to a palliative care approach. Determining when a condition becomes life-threatening is complex and depends on patient-specific factors such as age, disease-specific factors such as the stage of the disease, and clinical context or available resources. The SPICT-SA tool^72^ (see Box 4) can assist in identifying when a condition reaches this stage. Palliative care can be provided alongside curative treatments.

Practical steps for clinicians implementing a palliative care approach include:

- **Formation of a multidisciplinary team**: This team may include social workers, healthcare professionals, rehabilitation practitioners, doctors, and nurses.
- **Early communication with the patient and family**: Early in the disease, patients and their families should be well-informed regarding the diagnosis and prognosis.
- **Family involvement**: The family is an integral part of the management process and should be empowered through education and counselling, with family meetings from an early stage.
- **Advance care planning**: This is a critical component of the palliative care approach. This process provides an opportunity to explore the patient’s fears, goals of care, and future care needs. These should be documented but remain flexible and open to revision.
- **Management of physical symptoms:** Anticipation and treatment of physical symptoms are vital. Common symptoms include pain, weight loss, confusion, and constipation.^73^
- **Management of medications:** When treating elderly patients, medications, particularly opioids, must be initiated at low doses and increased gradually, to minimise side effects. For more information see Chapter 22 of the Standard Treatment Guidelines for Primary Health Care (Box 4).^35^
- **Addressing social concerns**: Social issues such as loneliness, future care and financial constraints must be discussed. Families should be screened for other vulnerable dependents who may rely on the patient.
- **Support for psychological well-being**: Psychological symptoms, including anxiety and fear, should be managed through social care, support groups, and open discussions about the patient’s concerns.
- **Provision of spiritual care**: Spiritual support should explore patients’ sense of meaning, purpose, and legacy.
- **Linking to resources**: Link patients and families to community resources such as Home Base Care organisations, Hospice and Dementia support (see Box 4).

### End-of-life care

End-of-life care is provided when patients are in their final weeks or days of life. At this stage, management should change, and an end-of-life care approach should be followed:

- Inform the family and patient that you are ‘concerned’ that the end ‘may’ be near. Avoid giving an exact timeframe.
- Rationalise all medications, such as statins (and possibly including ART), and discontinue interventions such as regular blood tests.
- Prescribe medication to address anticipatory symptoms, including pain, secretions, nausea, vomiting, and delirium.
- All interventions should prioritise comfort.
- Regular repositioning and oral care are essential.
- Nutrition should focus on comfort feeding, and the use of nasogastric tubes is not recommended.
- Provide the family with clear guidelines on what to do before, during and after the patient passes away.
- Link the family to bereavement support services.

## Conclusion

Ageing with HIV presents unique clinical challenges, including more comorbidities, polypharmacy, and an increased risk for geriatric syndromes such as frailty and cognitive impairment. These issues are further complicated by potential DDIs between ART and medications commonly used to manage age-related conditions. On the other hand, PWH are ‘in care’ and are relatively frequently encountered in the health system. Integrating geriatric assessments in a systematic and relatively pain-free manner may reduce unnecessary morbidity and improve ‘health span’ as well as ‘life span’ in OPWH. Integrating HIV care with geriatric principles can preserve functional independence and improve quality of life. A broader perspective of the needs of OPWH will be vital in ensuring that ageing PWH receive safe, effective, and compassionate care.

## Appendix 1

**TABLE 1-A1:** Cancer screening recommendations in older people with HIV.

**Breast cancer** Women over 40 years in the public sector: annual clinical breast examination, breast self-examination, awareness campaigns. Average risk women 40–75 years old who are asymptomatic in the private sector: mammogram, ultrasound or MRI as appropriate every 2nd year from age 40 years. Discontinue mammography screening for women older than 75 years. Women at high risk for breast cancer (e.g., family history): mammography screening at specialised breast clinics in both public and private sectors. **Cervical cancer** Cervical screening in the public sector depends on geographic location. Pap smear is used most frequently but liquid-based cytology (LBC) and Human papillomavirus (HPV)-based screening to be phased in. Sexually active women living with HIV regardless of age: ■ Conventional cervical cytology (Pap smear) every 3 years if last screening was normal. ■ Screen annually if last screening was abnormal. ■ Stop screening after 50 years if 2 consecutive negative screening tests. Areas with significant resource constraints still practice visual inspection with ascetic acid (VIA). **Prostate cancer** Men > 50 yrs old who are asymptomatic, with a life expectancy > 10 years: Prostatic-specific antigen (PSA) screening with intervals depending on the first PSA result. ■ Black African men or those with a first degree relative with prostate or breast cancer: commence PSA screening at 40–45 years. ■ Discontinue screening in asymptomatic men > 70 years. Digital rectal examination (DRE) only in symptomatic men or if PSA is elevated PSA reference range increases with age. PSA ≤ 4 ng/mL is acceptable. Defer PSA testing if prostatitis, acute urinary retention, urethral instrumentation or trans-urethral resection of the prostate (TURP). Repeat if PSA is elevated but < 10ng/mL in asymptomatic men with a normal DRE. Do free/total PSA to improve specificity of cancer diagnosis if PSA elevated but < 10 ng/mL. SA public sector: PSA screening is not routinely done; do PSA and DRE in symptomatic men. **Anal cancer** Men-who-have-sex-with-men (MSM) with HIV from 35 years, and men and women with HIV from 45 years: annual digital ano-rectal examination (DARE). International standard of care: DARE plus cytology and high-risk HPV testing. **Colorectal cancer** Average risk: screen from 45 years if life expectancy > 10 years and continue screening until 75 years. Faecal immunochemical test (FIT) has replaced faecal occult blood (FOB) test: to be done annually. ■ A positive FIT or FOB must be followed by colonoscopy. Screening colonoscopy intervals determined by findings at first colonoscopy. In the public sector colonoscopy screening is only offered at specialised units to individuals with a genetic predisposition or positive family history. **Hepatocellular cancer** Persons with cirrhosis: 6-monthly ultrasound of hepatobiliary system and serum alpha fetoprotein (AFP) at specialised units in public sector.

*Source:* Authors’ own compilation from: Wan Q, Anugwom C, Desalegn H, Debes JD. Hepatocellular carcinoma in Hepatitis B and human immunodeficiency virus coinfection in Africa: A focus on surveillance. Hepatoma Res. 2022;8:39. https://doi.org/10.20517/2394-5079.2022.32^67^, Sardanelli F, Aase HS, Álvarez M, et al. Position paper on screening for breast cancer by the European Society of Breast Imaging (EUSOBI) and 30 national breast radiology bodies from Austria, Belgium, Bosnia and Herzegovina, Bulgaria, Croatia, Czech Republic, Denmark, Estonia, Finland, France, Germany, Greece, Hungary, Iceland, Ireland, Italy, Israel, Lithuania, Moldova, The Netherlands, Norway, Poland, Portugal, Romania, Serbia, Slovakia, Spain, Sweden, Switzerland and Turkey. Eur J Radiol. 2017;27(7):2737–2743. https://doi.org/10.1007/s00330-016-4612-z^74^, Nicholson WK, Silverstein M, Wong JB, et al. Screening for breast cancer: US Preventive Services Task Force recommendation statement. JAMA. 2024;331(22):1918–1930. https://doi.org/10.1001/jama.2024.5534^75^, Nyström L, Bjurstam N, Jonsson H, Zackrisson S, Frisell J. Reduced breast cancer mortality after 20+ years of follow-up in the Swedish randomized controlled mammography trials in Malmö, Stockholm, and Göteborg. J Med Screen. 2017;24(1):34–42. https://doi.org/10.1177/0969141316648987^76^, Lauby-Secretan B, Scoccianti C, Loomis D, et al. Breast-cancer screening – Viewpoint of the IARC Working Group. New Engl J Med. 2015;372(24):2353–2358. https://doi.org/10.1056/NEJMsr1504363^77^, Cervical cancer prevention and control policy. National Department of Health of South Africa; 2017^78^, World Health Organization. Guideline for screening and treatment of cervical pre-cancer lesions for cervical cancer prevention. Geneva: World Health Organization; 2021^79^, Hall MT, Simms KT, Murray JM, et al. Benefits and harms of cervical screening, triage and treatment strategies in women living with HIV. Nat Med. 2023;29(12):3059–3066. https://doi.org/10.1038/s41591-023-02601-3^80^, John J, Adam A, Kaestner L, et al. The South African prostate cancer screening guidelines. S Afr Med J. 2024;114(5):15–18. https://doi.org/10.7196/SAMJ.2024.v114i5.2194^81^, Ilic D, Djulbegovic M, Jung JH, et al. Prostate cancer screening with prostate-specific antigen (PSA) test: A systematic review and meta-analysis. BMJ. 2018;362. https://doi.org/10.1136/bmj.k3519^82^, Stier EA, Clarke MA, Deshmukh AA, et al. International anal neoplasia society’s consensus guidelines for anal cancer screening. Int J Cancer. 2024;154(10):1694–1702. https://doi.org/10.1002/ijc.34850^83^, Jain S, Maque J, Galoosian A, Osuna-Garcia A, May FP. Optimal strategies for colorectal cancer screening. Curr Treat Options Oncol. 2022;23(4):474–493. https://doi.org/10.1007/s11864-022-00962-4^84^, Helsingen LM, Kalager M. Colorectal cancer screening – Approach, evidence, and future directions. NEJM Evidence. 2022;1(1):EVIDra2100035. https://doi.org/10.1056/EVIDra2100035^85^ and Villa E, Donghia R, Baldaccini V, et al. GALAD outperforms aMAP and ALBI for predicting HCC in patients with compensated advanced chronic liver disease: A 12-year prospective study. Hepatol Commun. 2023;7(10):e0262. https://doi.org/10.1097/HC9.0000000000000262^86^
